# Clinical impact of miR-223 expression in pediatric T-Cell lymphoblastic lymphoma

**DOI:** 10.18632/oncotarget.22386

**Published:** 2017-11-11

**Authors:** Elena Pomari, Federica Lovisa, Elisa Carraro, Simona Primerano, Emanuele S.G. D’Amore, Paolo Bonvini, Luca Lo Nigro, Rita De Vito, Luciana Vinti, Piero Farruggia, Marta Pillon, Giuseppe Basso, Katia Basso, Lara Mussolin

**Affiliations:** ^1^ Department of Women's and Children's Health, Clinic of Pediatric Hemato-Oncology, University of Padova, 35128 Padova, Italy; ^2^ Centre for Tropical Diseases, Ospedale Sacro Cuore-Don Calabria, 37024 Negrar, Italy; ^3^ Istituto di Ricerca Pediatrica, Fondazione Città della Speranza, 35127 Padova, Italy; ^4^ Institute of Pathology, San Bortolo Hospital, 36100 Vicenza, Italy; ^5^ Center of Paediatric Haematology, Azienda Policlinico-OVE, 95123 Catania, Italy; ^6^ Department of Paediatric Haemato-Oncology, IRCCS Ospedale Bambino Gesù, 00165 Roma, Italy; ^7^ Department of Paediatric Haemato-Oncology, ARNAS Ospedali Civico, G Di Cristina, 90127 Palermo, Italy; ^8^ Institute for Cancer Genetics, Department of Pathology and Cell Biology, Columbia University, NY 10027, New York, USA

**Keywords:** childhood, T-Cell lymphoblastic lymphoma, miR-223

## Abstract

Although probability of event-free survival in pediatric lymphoblastic T-cell lymphoma (T-LBL) is about 75%, survival in relapsed patients is very poor, so the identification of new molecular markers is crucial for treatment optimization. Here, we demonstrated that the over-expression of *miR-223* promotes tumor T-LBL cell growth, migration and invasion *in vitro*. We found out that SIK1, an anti-metastatic protein, is a direct target of *miR-223* and consequently is significantly reduced in *miR-223*-overexpressing tumor cells. We measured *miR-223* expression levels at diagnosis in tumor biopsies from 67 T-LBL pediatric patients for whom complete clinical and follow up data were available, and we found that high *miR-223* expression (above the median value) is associated with worse prognosis (PFS 66% vs 94%, P=0.0036). In addition, the multivariate analysis, conducted taking into account *miR-223* expression level and other molecular and clinical characteristics, showed that only high level of *miR-223* is an independent factor for worse prognosis. *MiR-223* represents a promising marker for treatment stratification in pediatric patients with T-LBL and we provide the first evidence of *miR-223* potential role as oncomir by SIK1 repression.

## INTRODUCTION

Childhood Lymphoblastic T-cell Lymphoma (T-LBL) represents about one-third of pediatric non-Hodgkin lymphomas (NHLs), and it originates from lymphoid progenitor cells arrested at early stages of T-cell maturation [1]. Patients with T-LBL often present with large mediastinal masses and generally no morphological bone marrow involvement. Indeed, since cell marker expression overlaps with that of T-lineage acute lymphoblastic leukemia (T-ALL), the clinical distinction between the two entities is arbitrarily determined by the degree of bone marrow involvement: patients with more than 25% of lymphoblasts in the bone marrow are classified as affected by T-ALL, whereas those with a lower degree of marrow replacement or no detectable lymphoblasts are classified as T-LBL patients [1]. Although the 5-year event-free survival (EFS) and overall (OS) survival for pediatric T-LBL patients substantially improved during the last decades, the prognosis of patients who suffered from a relapse remains poor [2, 3]. Furthermore, the intensive treatment regimens are accompanied by high toxicity with considerable mortality and morbidity, indicating a need for the identification of prognostic markers to allow early group stratification and design of risk-adjusted treatment protocols. Until now, 3 molecular markers have been reported in literature, which might be of prognostic relevance for pediatric T-LBL patients treated on BFM (Berlin-Munster-Frankfurt) based regimens: aberrations in chromosome 6q14-24, mutations of *NOTCH1* and/or *FBXW7* and mutations of *PTEN* [4]. In descriptive retrospective analyses of pediatric T-LBL patients, loss of heterozygosity (LOH) at chromosomal region 6q14-24 (LOH6q) has been shown to be significantly associated with adverse outcome and increased risk of relapse [2, 4]. In the largest study by Bonn *et al.*, LOH6q was observed in 12% of patients and the common deleted region and thus the region of main interest for the unfavorable prognosis was located at chromosome 6q16 [4]. Hotspot mutations in *NOTCH1* and/or *FBXW7* are observed in about 50% of pediatric T-ALL patients and reported to be associated with an improved treatment response or outcome [5, 6]. Concerning pediatric T-LBL patients, five studies were published dealing with *NOTCH1* and/or *FBXW7* mutations [4, 7, 8]. Bonn *et al.* observed *NOTCH1* mutations in 60% of patients and associated with a favorable prognosis [4]. Similar data were reported for pediatric patients with T-ALL treated according to the ALL-BFM protocol [9, 10], a comparable regimen to that of NHL-BFM group administered to T-LBL patients, suggesting that *NOTCH1* mutations might serve as a positive prognostic marker in the context of BFM-type treatment.

Comprehensive data about non-coding transcripts, such as microRNAs (miRNAs), are available for many hematological malignancies. We recently identified a miRNA expression profile specific for pediatric T-LBL [11], suggesting that few miRNAs, including *miR-223*, may play a major role in T-LBL pathogenesis. Over-expression of *miR-223* has been previously reported in T-ALL, where *miR-223* has been shown to promote the development of leukemia in a mouse model [12]. Moreover, FBXW7 has been identified as a main mediator of *miR-223* pro-oncogenic activity in T cells [13, 14]. These observations suggest that *miR-223* overexpression may provide an additional level of regulation to promote NOTCH1 signaling by repressing its negative modulator FBXW7. In the present study, we assessed for the first time the clinical and prognostic significance of *miR-223* in a large series of pediatric T-LBL cases and its correlation with *NOTCH1/FBXW7* mutational status and protein expression. Our data show that in patients with T-LBL *miR-223* has a prognostic value that appears to outweigh the prognostic value of *NOTCH1* mutations. In addition, our data suggest that the anti-metastatic SIK1 is a target of *miR-223* and over-expression of *miR-223* contributes to a more aggressive tumor phenotype.

## RESULTS

### Clinical features

To ensure that the study population with appropriate bioptic material was representative of the entire clinical cohort, we compared the EFS of the 67 analyzed patients with that of all the 114 patients enrolled in treatment protocols and no statistically significant differences were found (EFS= 78%, SE=5%, vs EFS=77%, SE= 4%, respectively, p=0.93) (Supplementary Table 1). The 67 patients with T-LBL evaluated for molecular markers had a median age of 9.3 years (range 1.1-16.6); most of them (89%) were diagnosed with disease at stage III-IV according to the St Jude's classification [15]; three of 67 had Central Nervous System (CNS) involvement. The main clinical characteristics of the 67 patients with T-LBL are listed in Table 1, along with the univariate and multivariate analyses to account for the variables of gender, stage of disease, age at diagnosis, CNS involvement, bone marrow involvement, mediastinal involvement, in addition to *NOTCH1* mutational status and *miR-223* expression level. The median follow-up of patients was 6.3 years (range: 0.7-14.5). Sixty-six (98.5%) of 67 patients reached complete remission during induction treatment. A total of 14 patients had a treatment failure due to: induction failure (n=1); death in first remission (n=1 as a result of septicemia); disease relapse (n=13; n=6 local, and n=7 local and new site), after a median time of 1.4 years from diagnosis (range 0.5-7.1 years). Of the 13 relapsed patients, only 3 are alive after autologous (n=1) or allogenic (n=2) hematopoietic stem-cell transplantation (HSCT), whereas 10 died as a result of disease progression despite second-line treatments.

**Table 1:** Clinical characteristics of the 67 patients with T-LBL

Characteristics	Categories	# Pts	Events	5-y PFS %(SE%)	UnivariateP-value	MultivariateP-value	Hazard ratio(95% CI)
**Median age**	≤ 9.5 yrs> 9.5 yrs	3334	68	81 (7)79 (7)	0.76	-	
**Gender**	MaleFemale	5116	113	78 (6)88 (8)	0.87	-	
**Stage**	I+IIIII+IV	760	113	86 (13)79 (5)	0.56	-	
**Mediastinal involvement**	YesNo	5215	95	82 (5)73 (11)	0.31	-	
**BM involvement**	YesNo	1057	212	80 (12)80 (5)	0.92	-	
**CNS involvement**	YesNo	364	212	82 (5)33 (27)	0.052	0.10	
***NOTCH1*** **mut**	NoYes	3235	113	68 (8)91 (5)	0.016	0.17	
***miR-223*** **expression level**	LowHigh	3433	212	94 (4)66 (8)	0.0036	0.012	6.8 (1.5-30.4)

Low= expression level ≤ median value (18).

High= expression level > median value (18).

BM= bone marrow.

CNS= Central Nervous System.

PFS=Progression-free survival.

Pts=patients.

SE=standard error.

CI=confidence interval.

### miR-223 is over-expressed in T-LBL cases

**Figure 1:**
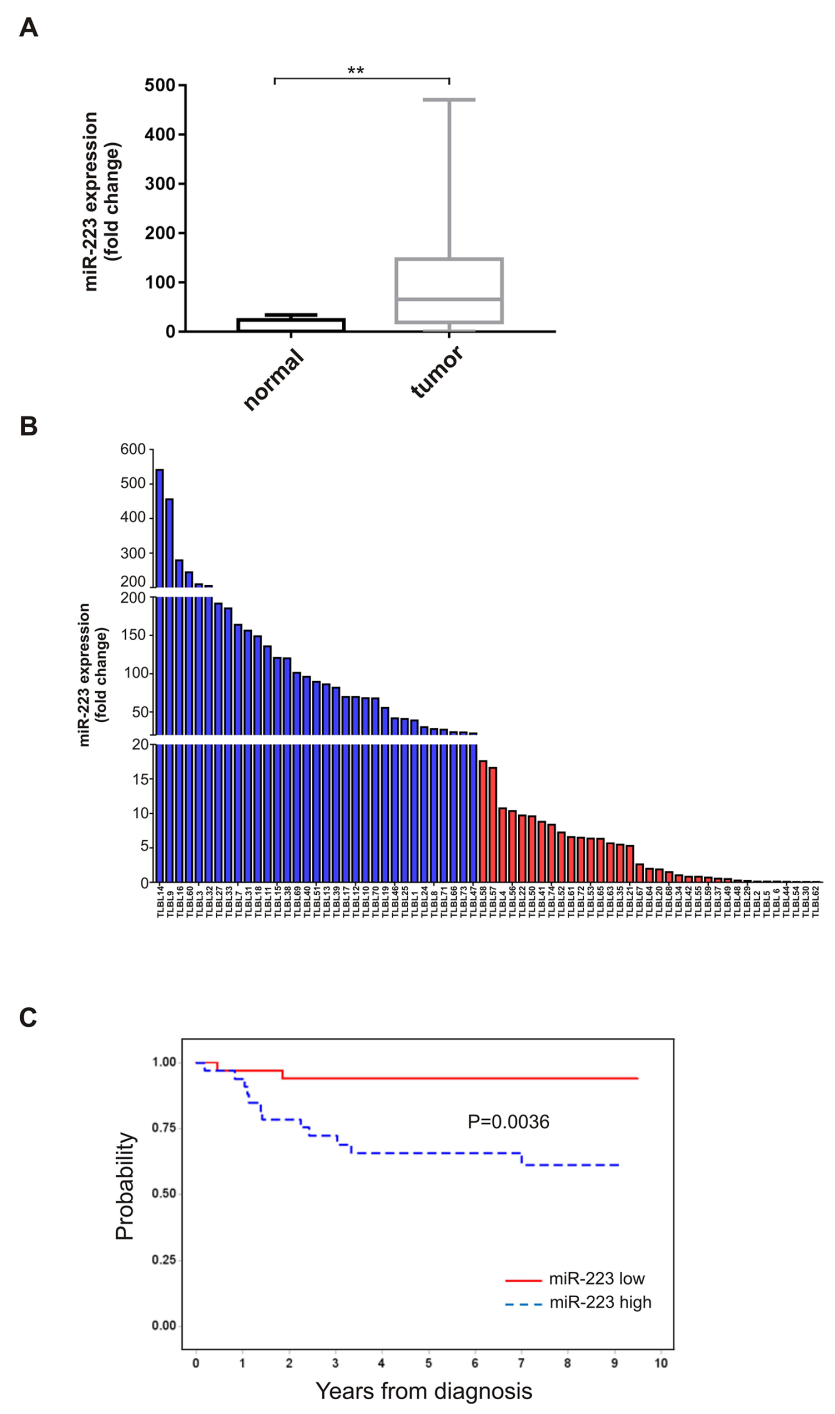
Expression levels of *miR-223* in T-LBL patients **(A)** Boxplot of differential *miR-223* expression in T-LBL tumour biopsies (n=67) and in normal thymus tissue (n=5) detected by qRT-PCR (^**^P=0.003). Data have been calculated according to the comparative delta Ct method (2^-ΔΔCt^), using RNU6 as endogenous control. The horizontal line in the box indicates the median expression level of *miR-223.* **(B)** Heterogeneous expression levels of *miR-223* in 67 T-LBL cases. Data have been measured by qRT-PCR according to the comparative delta Ct method (2^-ΔΔCt^), and compared to normal thymus tissue. Blue and red bars are T-LBL tumor biopsies with higher (> median value) and lower (≤ median value) level of *miR-223*, respectively. *MiR-223* median expression level=18. **(C)** PFS according to *miR-223* expression level. *MiR-223* high (>median value), *miR-223* low (≤median value).

We previously defined a T-LBL specific miRNA signature that includes *miR-223* as specifically over-expressed in T-LBLs compared to their normal counterpart [11]. Here, we confirmed our previous observation in 67 T-LBL cases by qRT-PCR analysis. Indeed, *miR-223* was up-regulated up to 400 times compared to normal thymus tissue (Figure 1A). Interestingly, the expression levels of this miRNA in T-LBL patients displayed a heterogeneous distribution (Figure 1B). In order to evaluate the prognostic impact of miR-223 expression, we defined two groups of patients that express high (above the median value) or low (equal or below the median value) levels of miR-223, respectively. The results showed that *miR-223* high level was associated with worse prognosis, with a progression-free survival (PFS) of 66% (SE= 8%) for high expressing cases vs 94% (SE= 4%) for low expressing (P = 0.0036, Figure 1C).

### NOTCH1 activation is independent from miR-223 in T-LBL

**Figure 2:**
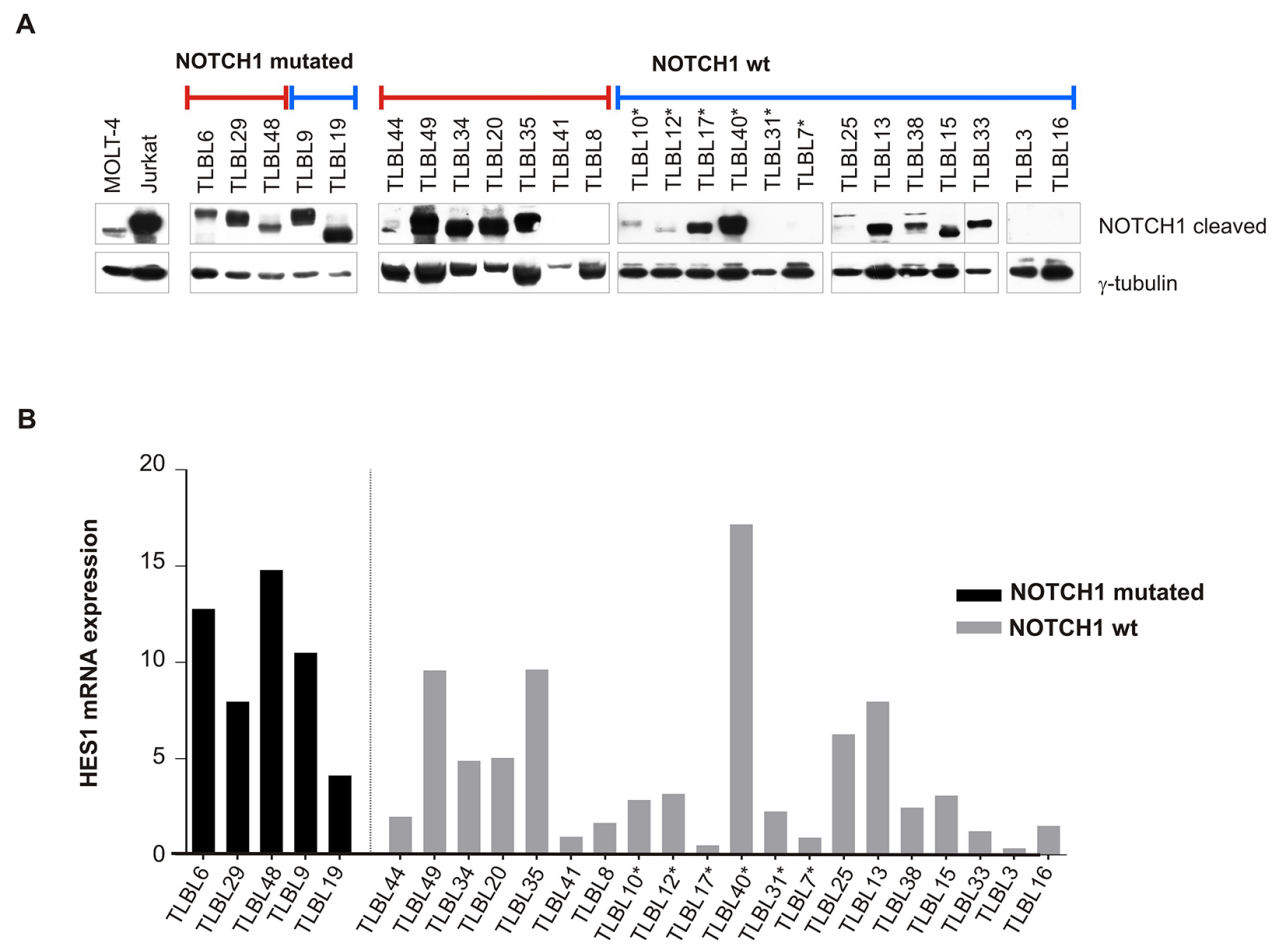
NOTCH1 pathway activation in T-LBL primary tumors **(A)** Western blotting analysis of intracellular cleaved NOTCH1 in T-LBL tumour biopsies. Blue line: patients with *miR-223* high expression level; red line: patients with *miR-223* low expression level. Lysates from cell lines MOLT-4 and Jurkat were included as positive controls. γ-tubulin was used as loading control. Vertical lines have been inserted to indicate repositioned gel lanes. **(B)** Expression levels of HES1 mRNA in primary tumors measured by qRT-PCR. Data have been calculated according to the comparative delta Ct method (2^-ΔΔCt^), using ABL gene as endogenous control. Relapsed patients are indicated with ^*^.

Over-expression of *miR-223* has been previously reported in T-ALL, where it has been shown to regulate NOTCH1 signaling by repressing its negative regulator FBXW7 [14]. In this context, constitutive activation of this signaling pathway is further determined by gain of function mutations of *NOTCH1* and inactivating mutations of *FBXW7* [9, 10]. To evaluate the activation of NOTCH1 pathway in T-LBL, we first analyzed *NOTCH1/FBXW7* mutational status in all 67 patients. In addition, the expression of active intracellular cleaved NOTCH1 (ICN1) was assessed in 25/67 patients with bioptic material available for immunoblotting analysis. *NOTCH1* mutations were found in 35/67 T-LBL patients (52%), with 28 patients having gain-of-function mutations in HD domain and 18 in PEST domain, respectively (Supplementary Table 2). In 11 patients, mutations were detected in both the HD and PEST domains. Among HD mutations, 20/28 were single amino acid substitutions and 8/28 were in-frame insertions or deletions (indels) (Supplementary Figure 1). PEST-domain truncating mutations were observed in 11/18 cases. Of them, 4/11 were non-sense mutations while the other 7 were out-of-frame indels. PEST missense mutations were detected in 6/18 patients. The remaining patient (TLBL74) had a short in-frame insertion (Supplementary Figure 1). Point mutations in *FBXW7* hot-spot exons were detected in 14/67 patients (data not shown). These data are consistent with previous reports on pediatric T-LBL [4, 7, 8, 13]. The active cleaved form ICN1 was found expressed in all 5 *NOTCH1* mutated cases and in 14/20 cases negative for mutations in HD and PEST domains (Figure 2A). The activation of NOTCH1 signaling pathway was confirmed by measuring the transcript levels of its direct target HES1 by qRT-PCR (Figure 2B). As expected, HES1 transcript levels significantly correlated with ICN1 protein expression (Spearman's correlation coefficient r=0.5777, P<0.05). However, the expression level of HES1 was not significantly associated to relapse/progression events (Fisher's exact test, P=0.69). Moreover, no significant difference was observed between *miR-223* levels and ICN1 expression in tumor biopsies (Fisher's exact test, P=0.36), suggesting that NOTCH1 activation is independent from *miR-223* expression in lymphoma.

### Prognostic significance of miR-223 expression overcomes NOTCH1/FBXW7 mutational status

**Figure 3:**
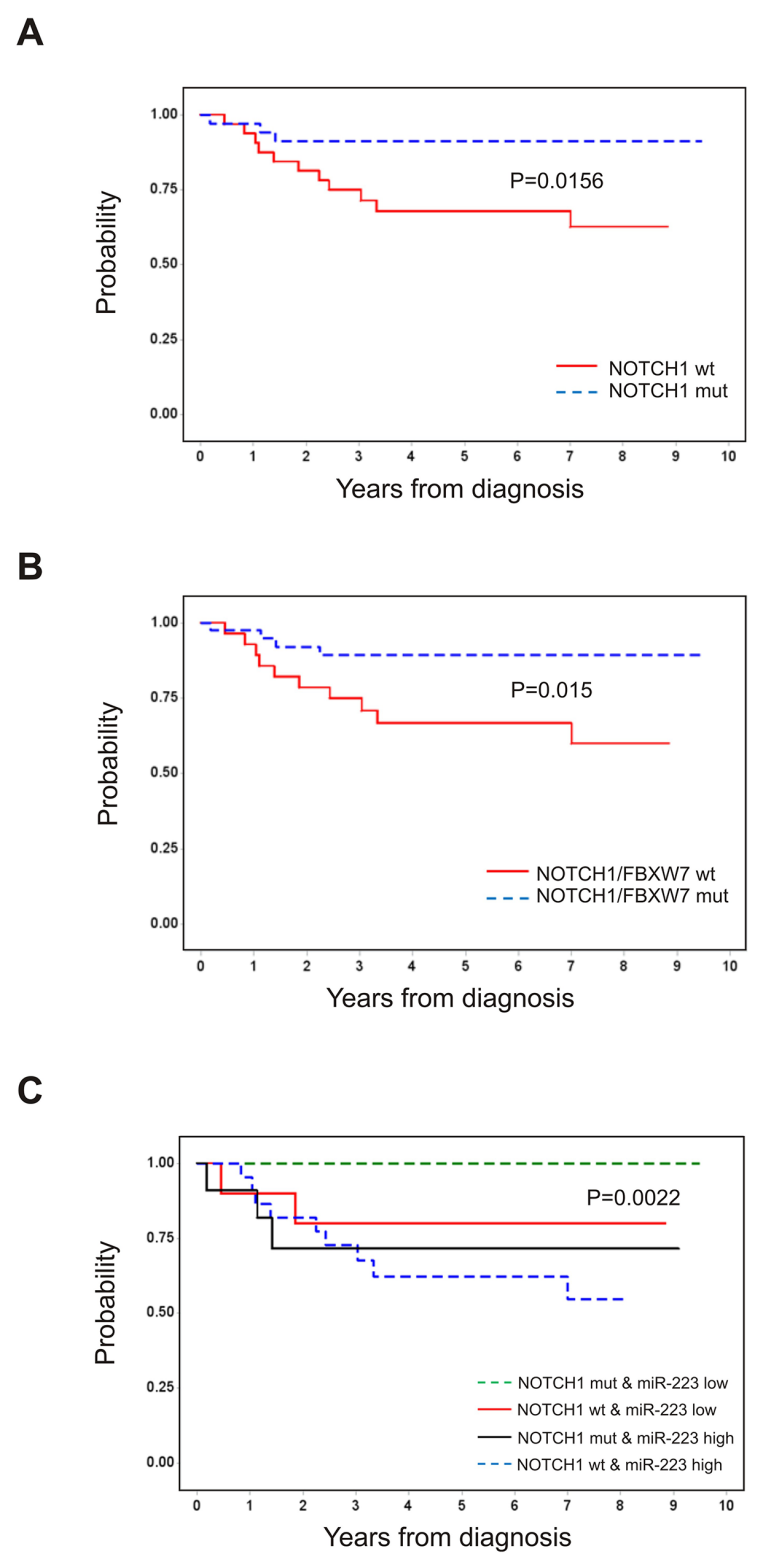
Prognostic significance of *NOTCH1* and *FBXW7* mutational status and *miR-223* expression level Outcome of 67 pediatric patients with T-LBL stratified by *NOTCH1* and *FBXW7* mutational status. PFS according to mutation of *NOTCH1* **(A)**, *NOTCH1* and/or *FBXW7* **(B)**. **(C)** PFS according to mutation of *NOTCH1* and *miR-223* expression level. MUT, mutated; WT, wild type. *MiR-223* high (>median value), *miR-223* low (≤median value).

To define the prognostic impact of *NOTCH1* mutations in T-LBL, patients were grouped according to *NOTCH1* and *FBXW7* mutational status. As reported in other studies [4], *NOTCH1*^mut^ patients showed significantly better outcome than *NOTCH1*^wt^, with 91% (SE= 5%) vs 68% (SE= 8%) 5-year PFS, respectively (P = 0.0156; Figure 3A). The trend was also significant when *NOTCH1/FBXW7* mutational status was evaluated, showing 89% PFS (SE, 5) for mutated vs 67% (SE= 9%) for unmutated cases (P = 0.015, Figure 3B), respectively. No significant difference was found considering *FBXW7* mutational status alone (P = 0.47, data not shown), in line with data previously reported [4]. To further assess the role of *miR-223* in the outcome of disease, we combined *miR-223* expression with *NOTCH1* mutational status. PFS of patients carrying *NOTCH1*^wt^ and elevated *miR-223* was 63% (SE= 10%) vs 80% (SE= 13%) for patients NOTCH1^wt^ with low *miR-223* (p=0.006). In contrast, patients with *NOTCH1*^mut^ and elevated *miR-223* showed a significant inferior PFS compare to those with *NOTCH1*^mut^ with low level of *miR-223 (*72% SE, 14 vs 100%) (P = 0.016, Figure 3C). These findings suggested that *miR-223* expression might outweigh the prognostic value of *NOTCH1* mutations in T-LBL. Indeed, in multivariate analysis *miR-223* expression level resulted the only significant negative prognostic parameter (P=0.012) (Table 1).

### miR-223 involvement in tumor cell growth, migration and invasion

**Figure 4:**
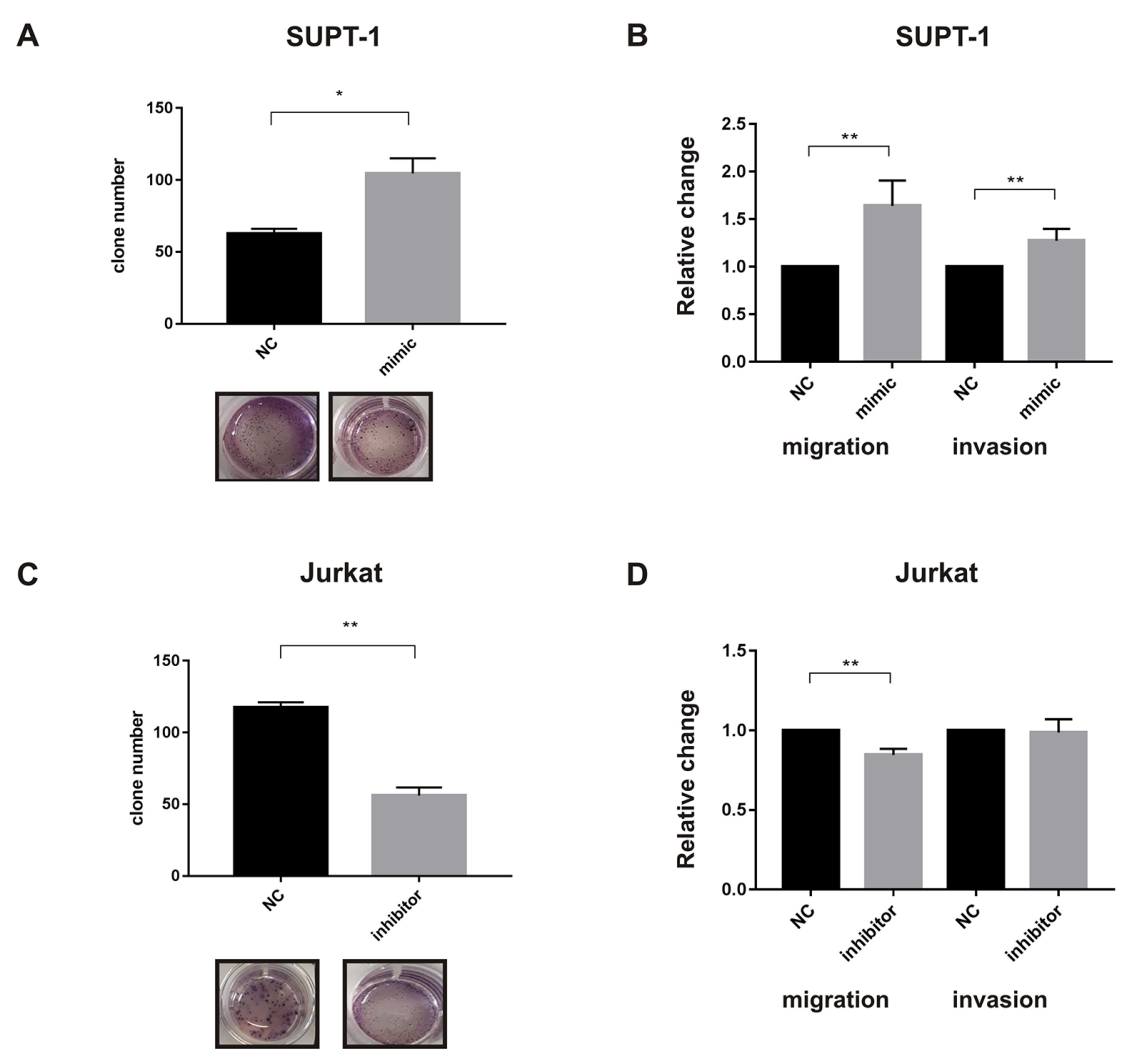
*miR-223* involvement in tumor cell growth, migration and invasion SUPT-1 cells that express low levels of *miR-223* were transiently transfected with *miR-223* precursor (mimic) or its negative control (NC) and used to analyze clonogenicity **(A)** or migration and invasion **(B)**. Conversely, Jurkat cells, that expresses high level of *miR-223*, were transiently transfected with inhibitor (anti-miR or anti-control) and used to analyze clonogenicity **(C)** or migration and invasion **(D)**. The results are shown as number of colonies measured at 10 days by colony assay (A-C), and percentage (%) of migration and invasion by Transwell-migrating or Matrigel-invading cells (B-D). Clonogenicity, migration and invasion of miR-modulated cells are shown relative to negative control values. Two or three independent experiments were performed in triplicate and mean results are shown. ^*^P<0.05; ^**^P<0.001.

To further elucidate the functional role of *miR-223* in lymphomagenesis, we evaluated its effect on cell behavior *in vitro*, by transiently over-expressing *miR-223* in the T-LBL cell line SUPT-1 and inducing its inhibition in the T-ALL cell line Jurkat. We choose these cell lines for their low and high endogenous expression level of *miR-223*, respectively [14] (Supplementary Figure 2). *MiR-223* expression level was evaluated by qRT-PCR at different time points after transfection (Supplementary Figure 3). MTT assay showed an increase of cell growth to 25% in SUPT-1 cells at 72 h, while a 20% reduction in Jurkat cells (data not shown). In addition, we observed an increase of clonogenic growth capacity in SUPT-1 over-expressing *miR-223* (Figure 4A). Interestingly, *miR-223* over-expressed in SUP-T1 incremented significantly migration and invasion compared to control cells (Figure 4B). Consistently, the inhibition of *miR-223* in Jurkat cells led to a reduction in clonogenicity and motility after transfection with *miR-223* inhibitor compared to control (Figure 4C and 4D). Taken together, these results suggest that *miR-223* is involved in tumorigenic processes of lymphoma and contribute in promoting cell aggressiveness.

### miR-223 down-regulates the expression of SIK1 and contributes to a more aggressive phenotype in pediatric T-LBL patients

**Figure 5:**
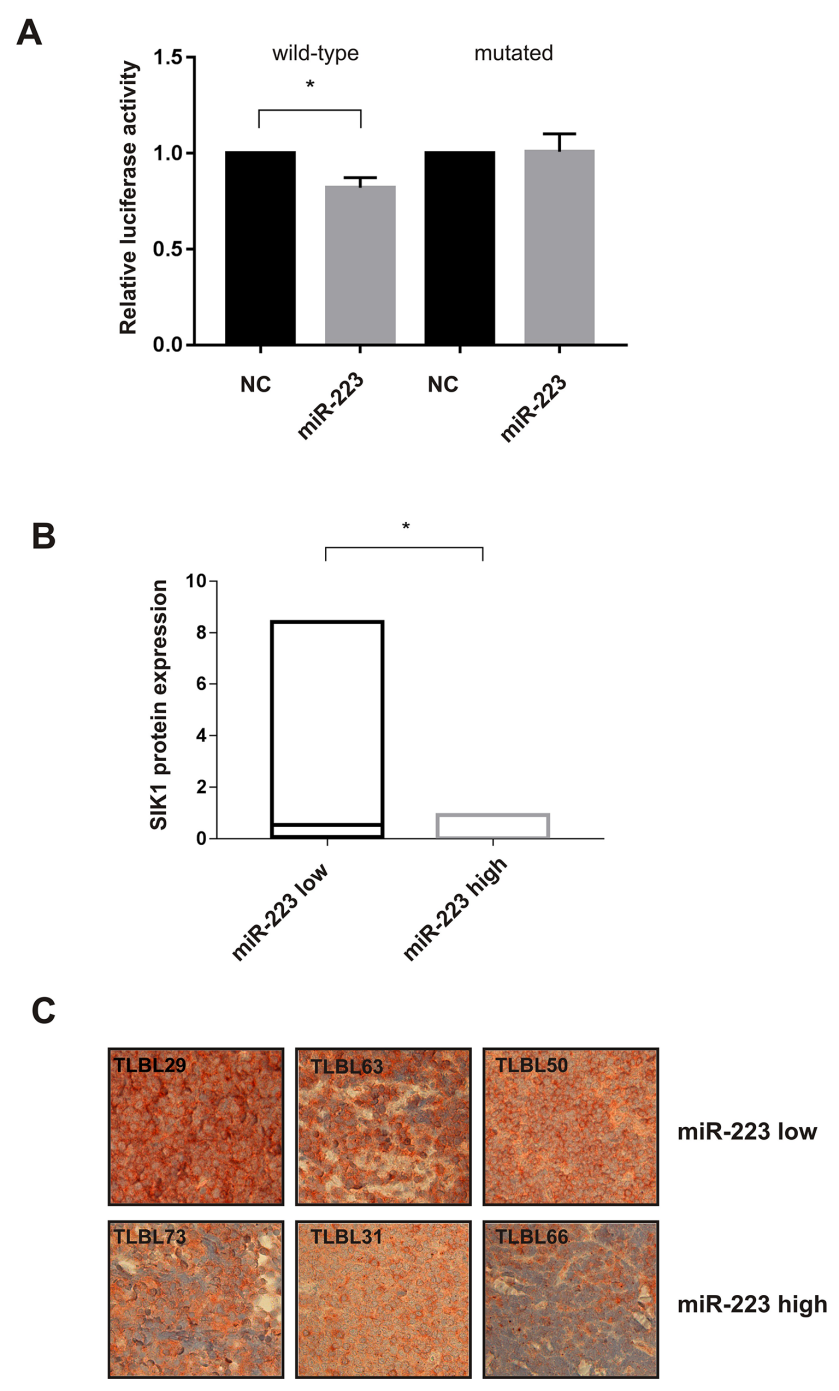
*miR-223* modulates the expression of SIK1 SUPT-1 cells transfected with *miR-223* precursor analyzed 24 h post-transfection were used to perform Luciferase assay in cells transfected with reporter containing wild-type or mutant (mutated) binding sites in *SIK1* 3’-UTRs. Results from three independent experiments are shown as mean ± SEM of Firefly luciferase activity relative to controls (NC), normalized on Renilla luciferase activity. ^*^P<0.05, **(A)**. Boxplot represents the analysis of SIK1 protein expression in T-LBL tumour biopsies detected by immunoblotting. Density expression level was normalized on tubulin. The horizontal line in the box indicates the median expression level of SIK1 in the two *miR-223* subgroups (*miR-223*-low and -high) of T-LBL cases. ^*^P<0.05 **(B)**. SIK1 staining in T-LBL specimens, belong to *miR-223*-low (T-LBL_29,63,50) and -high groups (T-LBL_73,66,31) (× 40) **(C)**.

Based on a previous analysis that we performed to identify context-specific miRNA targets in T-LBL by integrating computational predictions of miRNA targets with T-LBL microRNA and gene expression data [11], *SIK1* (Salt-inducible kinase 1) emerged as a strong candidate to be a direct target of *miR-223* in T-LBL (P <0.0001). SIK1 is an important regulator of anoikis and its loss has been shown to facilitate metastatic spread and survival of disseminated cells as micrometastases in solid tissues such as lungs [16]. Thus, by using the bioinformatics tool MicroRNA.org, we found that *SIK1* 3’-UTR is characterized by two putative binding sites for *miR-223* in the up- and down-stream regions that we named UTR-1 and UTR-4, respectively (Supplementary Figure 4). Direct modulation by *miR-223* of *SIK1* was confirmed by 3’UTR luciferase assay in SUPT-1 T-LBL cell line transfected with *miR-223* or negative control and reporter vectors containing the entire 3’-UTR of *SIK1* divided in 4 portions, namely UTR-1, 2, 3 and 4. We observed a significant reduction (20%) in luciferase activity in construct containing the UTR-4 portion of *SIK1* 3’-UTR, an effect that was abrogated by point mutations in the *miR-223* interacting region of the 3’-UTR of *SIK1* (Figure 5A). No significant effect was observed in UTR-1 (data not shown). Moreover, no repression was seen in cells transfected with portions UTR-2 and UTR-3, confirming that these sequences are not directly targeted by *miR-223* (data not shown). In support of the modulation of *SIK1* by *miR-223*, we found a marked decrease of SIK1 protein expression in *miR-223* over-expressing SUP-T1 cell line up to 72h post-transfection, when analyzed by immunoblotting analysis (Supplementary Figure 3). This evidence was not observed in Jurkat cells after inhibition of *miR-223*. Thus, we examined SIK1 protein expression in available tumour biopsies by immunoblotting (Supplementary Figure 5). Interestingly, the results showed that SIK1 protein level was significantly decreased in patients expressing high levels of *miR-223* (Figure 5B). Notably, in all relapsed patients *miR-223* was high and SIK1 protein was almost undetectable. Furthermore, when tumour biopsies were assessed for protein expression by immunohistochemistry, SIK1 was found weakly expressed in high *miR-223* cases (Figure 5C), suggesting that high levels of *miR-223* could function as oncomir by SIK1 repression.

## DISCUSSION

Identification of biological markers that can predict outcome in T-LBL is crucial to allow for treatment optimization and may serve as targets for new treatment strategies. Several recent studies have revealed that microRNAs are involved in the development of tumors [17]. The biologic role of microRNAs may vary according to their expression in distinct cell populations of normal or neoplastic tissues. Here, we report that expression levels of *miR-223* constitute a strong prognostic factor in pediatric T-LBL patients homogeneously treated according to BFM-type strategy. Indeed, we showed that high levels of *miR-223* expression are associated with worse prognosis. This study demonstrates, for the first time, that a single noncoding RNA associates with clinical outcome in T-LBL, in the context of the well-established molecular marker *NOTCH1*. Of note, when we combined *miR-223* expression levels and *NOTCH1* mutational status, we observed that *miR-223* outweighs the prognostic value of *NOTCH1*. Existing evidence in T-ALL showed *miR-223* as promoter in the development of leukemia [12] and it was demonstrated that over-expression of *miR-223* is an additional way of NOTCH1 signaling activation [18]. Our data suggest that in T-LBL, unlike T-ALL, *miR-223* expression and NOTCH1 pathway activation are not correlated. Indeed, both T-LBL and T-ALL represent T-cell progenitor malignancies, that share overlapping clinical, morphological and immunophenotypic features, however comparative genomic studies revealed that, despite having some genetic similarities, these two manifestations of T-cell malignancy may reflect distinct biological entity [19, 20]. Our results corroborate these data, highlighting that *miR-223* over-expression affects the aggressiveness of the disease. Based on miRNA-target predictions, we found SIK1 protein as a putative target of *miR-223*. SIK1 kinase has been identified as a key modulator of anoikis (apoptosis induced by cell detachment) and its inactivation compromised the tumor suppressor p53 function, allowing metastatic growth in transplanted tumor cells [21]. Reduced SIK1 expression is correlated with poor prognosis in two large human breast cancer data sets [16]. No data are available regarding SIK1 expression in pediatric tumors and in particular in T-LBL. Here we demonstrated that SIK1 is one of the targets of *miR-223*. Consistent with the findings of previous studies reporting that SIK1 prevents metastasis and tumor invasion [16], we found that SIK1 can be down-regulated in T-LBL tumour tissues, suggesting its association with the T-LBL disease progression and its potential use as predictor marker for patient risk stratification at diagnosis. Indeed, we found a significant different expression level of SIK1 protein in T-LBL cases based on *miR-223* expression level. Noteworthy, all relapsed patients displayed high level of *miR-223* and very low level of SIK1 at diagnosis. Based on our multivariate analysis, the negative prognostic power of *miR-223* could be considered to identify high risk patients that could be treated with an intensification of current therapies. *MiR-223* expression level analysis in tumour tissue of a larger cohort of patients will contribute to confirm the importance of this molecular marker for risk-based treatment stratification in T-LBL.

## MATERIALS AND METHODS

### Patients and methods

Between June 2000 and May 2012, 114 consecutive children with T-LBL were enrolled in an NHL-BFM-type treatment protocols (AIEOP LNH-97 or EuroLB-02) [7, 22]. Of these, 67 patients had available tumor specimens for both *NOTCH1/FBXW7* mutational analysis and *miR-223* expression level evaluation. The availability of tumor specimens was the only selection criteria. The infantile normal thymuses had been removed during cardiothoracic surgical procedures and were obtained from our hospital. Protocols were approved by the ethics committee or by the internal review board of each participating institution, and inform consent was obtained from parents or legal guardians before patient enrollment. The diagnosis of T-LBL was established from clinical, histological, and immunohistochemistry findings. Tumor biopsies were classified according to WHO guidelines [23]. In all cases, the histological diagnosis was centrally reviewed. Samples were collected in accordance with the tenets of the Declaration of Helsinki. Ethics approval and consent to participate this study was approved by the Ethics Committee, Padua Hospital. Written informed consent was obtained from all subjects.

### NOTCH1 and FBXW7 mutational analysis

Genomic DNA was obtained from tumor tissue biopsies or pleural/pericardial effusion of T-LBL cases, as described previously [24]. Exons 26 and 27, encoding for *NOTCH1* heterodimerization domain (HD), exon 34, encoding the transactivation domain (TAD) and PEST domains, and *FBXW7* exons 9, 10 and 12 were amplified as previously reported [4]. PCR products were sequenced on a 3500 DX Genetic Analyzer (Life Technologies, CA, USA), either directly or after subcloning into Topo TA cloning vector (Life Technologies). GeneBank accession numbers NM_017617.3 and NM_033632.3 were used as reference sequences.

### qRT-PCR for miRNA or mRNA detection

Total RNA was isolated using Trizol Reagent (Life Technologies), as described previously [25]. Validation of miRNA detection for *in vitro* experiments was performed using TaqMan® MicroRNA Reverse Transcription kit and TaqMan® Universal PCR Master Mix No Amperase® UNG (Life Technologies). Three replicates of each sample and endogenous control were amplified for each real-time PCR (qRT-PCR) reaction. For mRNA detection, 1 μg of total RNA was retrotranscribed with SuperScript II reverse transcriptase (Life Technologies) and random hexamers, and qRT-PCR was carried out using TaqMan® Universal PCR Master Mix (Life Technologies). Each sample was analyzed in triplicate. The expression levels of specific targets of interest were assessed using hsa-*miR-223* (ID 002295), HES1 (ID 00172878_m1) (Life Technologies). RNU6B small nuclear RNA (ID 001093) (Life Technologies) or ABL [11] were chosen, as endogenous normalizers of the expression of miRNA or mRNAs, respectively. The relative expression levels between samples were calculated using the comparative delta Ct (threshold cycle number) method (2^-ΔΔCt^).

### Immunoblotting analysis

Sample lysates were prepared by SDS-buffer (1% SDS; 1 mM PMSF; 20 μg/mL leupeptin; 20 μg/mL aprotinin; 1:100 phosphatase inhibitor cocktails 2 and 3) to dissolve the pellet before SDS-PAGE analysis. Protein concentration was determined by BCA protein assay (Thermo Scientific Pierce, Milano, Italy), and equal amounts of proteins (50 μg) were resolved by SDS-PAGE prior to be transferred onto nitrocellulose membranes (PerkinElmer, MA, USA). Blocked membranes were probed for Notch1^Val1744^ (rabbit, 1:1000, Cell Signaling, MA, USA) and SIK1 (Y-20) (rabbit, 1:2000, Santa Cruz, CA, USA), using γ-tubulin (mouse, 1:4000, SIGMA, Milan, Italy) as gel loading control. Membranes were incubated with peroxidase-labeled donkey anti-rabbit IgG (1:2000, GE Healthcare, UK) or peroxidase-labeled sheep anti-mouse IgG (1:2000, GE Healthcare, UK) and visualized with ECL Pro chemiluminescent solution (PerkinElmer, MA, USA) and Hyperfilm autoradiography films (GE Healthcare, UK).

### Cell culture

Human cell lines SUPT-1 (NIBSC, UK) and Jurkat (ATCC, USA), were maintained in RPMI 1640 medium contained 10% heat-inactivated fetal calf serum (FCS), 2 mmol/L glutamine, 100 U/mL penicillin, and 100 μg/mL streptomycin at 37°C in 5% CO_2_ in a humidified incubator. All reagents were purchased from Life Technologies.

### Transient transfection of pre-miRNA or anti-miRNA

Pre-miRNA negative control (EX479903001), anti-miRNA negative control (EX199006101, power inhibitor control), miRNA precursor (EX47XXXX001) and inhibitor (EX410XXX101) hsa-*miR*-*223*-*3p* were purchased from Exiqon (Exiqon miRCURY LNA, Denmark). To induce transient pre-miR or anti-miR expression, SUPT-1 and Jurkat cells were transfected with 50 nM pre-*miR-223*/control or with 100 nM anti-*miR-223*/control respectively, using Amaxa® Cell Line Nucleofector® Kit V (Lonza, Visp, CH) following the manufacturer's instructions. Cells were harvested 24 h after transfection and miR-223 expression was assessed by qRT-PCR.

### MTT assay

SUP-T1 and Jurkat cell growth was assessed by MTT (3-4,5-dimethylthiazol-2,5-diphenyltetrazolium; SIGMA, Milan, Italy). The cells were grown after transfection up to 72 h and MTT salt reduction was measured each day at a 540 nm optical density (O.D.). Values were calculated as mean ±SD of triplicate cultures of three independent experiments.

### Migration, invasion and clonogenic assays

Migration was tested using cell culture inserts (Transwell) with a 5-μm pore size membrane (24-well format; Corning, MA, USA). Invasion was measured using 5-μm pore polycarbonate filter coated with Matrigel (Becton Dickinson, NJ, USA). The lower compartment contained 0.5 ml of 10% serum medium conditioned by 80 ng CXCL12 (Peprotech, London, UK) as a chemoattractant or serum-free RPMI medium as a control. In the upper compartment, 3×10^5^ SUPT-1 or Jurkat cells per well were placed in triplicate and incubated up to desired time point at 37°C in a humidified incubator with a 5% CO^2^ atmosphere. After incubation, the cells on the lower surface were counted. For clonogenic assay, SUPT-1 or Jurkat cells were incubated in the medium MethoCult™ H4230 (STEMCELL™, Hong Kong) at a cell density of 2×10^4^ cells/ml for 10 days. For all the assays, cells were previously transfected with mimic or inhibitor of *miR*-*223* and relative negative controls. Numbers of colonies were counted according to the manufacturer's instructions.

### Luciferase reporter assay

The 3’-UTR region of the predicted *miR-223* target gene *SIK1* was amplified from human genomic DNA and cloned into the pmirGLO Dual-Luciferase miRNA Target Expression Vector (Promega, Milan, Italy). To confirm the binding of *miR-223* to the 3’-UTR sequence of *SIK1*, SUPT-1 cells (1×10^6^) were co-transfected with 50 ng of the pmirGLO dual-luciferase constructs containing the 3’-UTR of *SIK1* and 50 nM of pre-*miR-223* or miRNA negative control. Lysates were collected 24 h after transfection and Firefly and Renilla Luciferase activities were measured with a Dual-Luciferase Reporter System (Promega, Milan, Italy). Relative luciferase activity was calculated by normalizing the ratio of Firefly/Renilla luciferase to negative control-transfected cells. Transfections were performed in triplicate. Moreover, the 3’-UTR construct was mutagenized at the *miR-223* binding site using the Phusion Site-Directed mutagenesis kit (Thermo Fisher Scientific, MA, USA) following the manufacturer's instructions.

### Immunohistochemistry

SIK1 protein expression was tested by immunohistochemistry on 6 formalin-fixed paraffin-embedded T-LBL tumor specimens using the SIK1 (Y-20) antibody (1:200 dilution, Santa Cruz), after deparaffinisation and antigen retrieval. Detection was performed using a biotinylated secondary antibody and a DAB chromogen in haematoxylin counterstained cells (Vectastain ABC Kit Elite, PK-6100, Vector Labs, Burlingame, CA, USA). Each case was considered positive when more than 50% of the cells had moderate-to-strong cytoplasmic staining.

### Statistical analyses

Data analysis were carried out by using the SAS statistical program (SAS-PC, version 9.3; SAS Institute Inc., Cary, NC, USA) and GraphPad Prism 7.0. Probability of progression-free survival (PFS) was analyzed by the Kaplan–Meier method and difference among patient subsets compared by log-rank test [26]. PFS was calculated from the date of diagnosis to the date of progression or relapse or to the date of the last follow-up. Univariate comparisons were performed according to the log-rank test, whereas the Cox model [27] was performed to examine the risk factors affecting the PFS in the multivariate analyses, including variable with *P*<0.2 in univariate analysis. Descriptive statistics were used to define population. The Spearman's correlation coefficient was used to measure the association between active intracellular cleaved NOTCH1 expression and HES1 transcript levels. The Fisher's exact test was used to evaluate the association among NOTCH1 activation and *miR-223* expression. The t-test was used to determine differences in cell growth, migratory and invasion potential and luciferase activity between different experimental conditions. A P-value less than 0.05 was considered statistically significant. All P-values are two-sided, with a type I error rate fixed at 0.05.

## SUPPLEMENTARY MATERIALS FIGURES AND TABLES







## Abbreviations

3′-UTR: 3′-untranslated region
BFM: Berlin-Munster-Frankfurt
BM: bone marrow
CNS: central nervous system
EFS: event-free survival
HD: heterodimerization domain
ICN1: active intracellular cleaved NOTCH1
LOH: loss of heterozygosity
miRNAs: microRNAs
NHLs: non Hodgkin lymphomas
OS: overall survival
PFS: progression-free survival
qRT-PCR: quantitative real-time PCR
TAD: transactivation domain
T-ALL: T-lineage acute lymphoblastic leukemia
T-LBL: T-cell lymphoblastic lymphoma
wt: wild-type
