# Supplementary material for: Clinical impact of miR-223 expression in pediatric T-Cell lymphoblastic lymphoma
Source: Oncotarget. 2017 Nov 11;8(64):107886–98. doi: 10.18632/oncotarget.22386 (PMC5746112; doi:10.18632/oncotarget.22386)
Supplement: Supplementary file 1 [file oncotarget-08-107886-s001.pdf]

Clinical impact of miR-223 expression in pediatric T-Cell lymphoblastic lymphoma

SUPPLEMENTARY MATERIALS

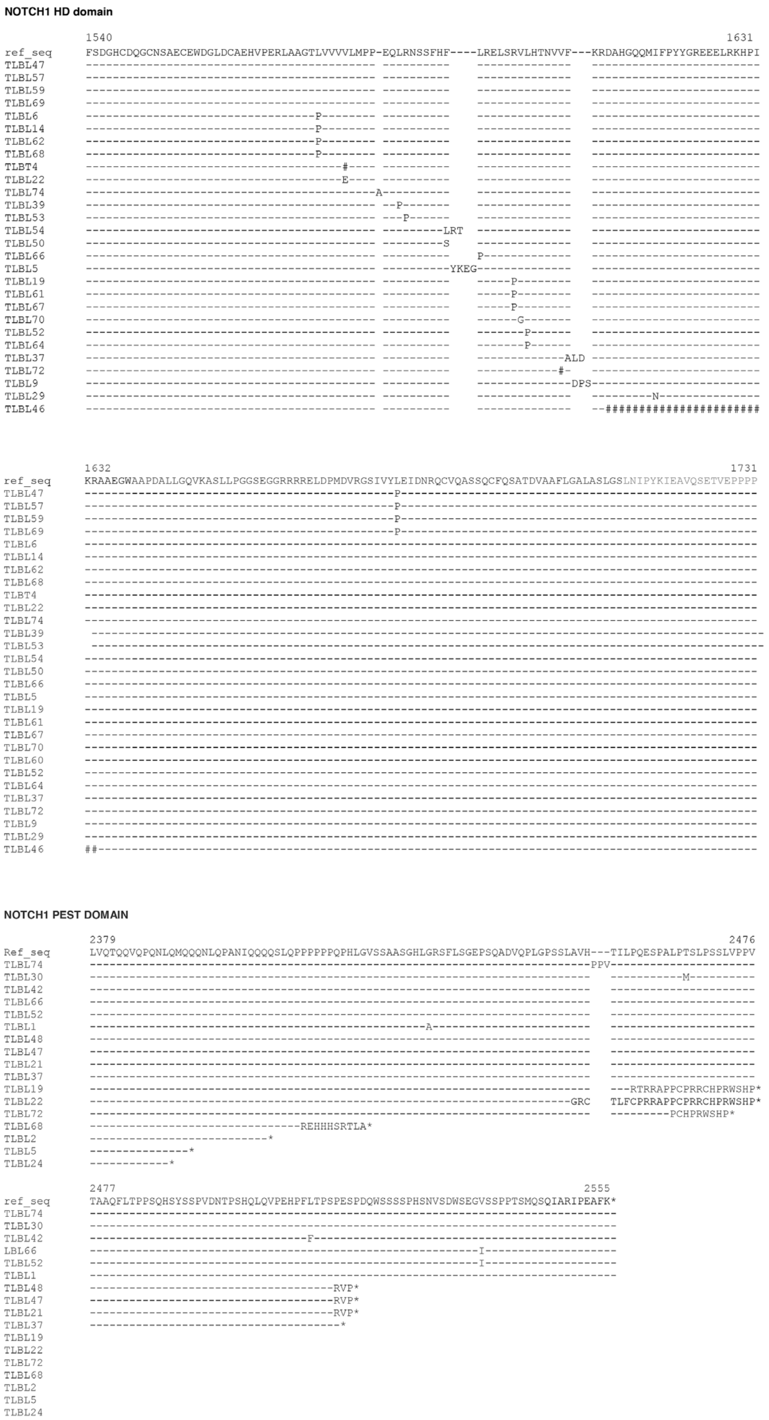

Supplementary Figure 1: Multiple sequence alignment of NOTCH1 wild-type and variants identified in the present study. Number of first and last depicted amino acids of reference sequence (ref_seq) are indicated based on NP_060087.3. Sequence differences caused by missense mutations, insertions or frame-shifts are shown. * premature stop codon, # deleted position.

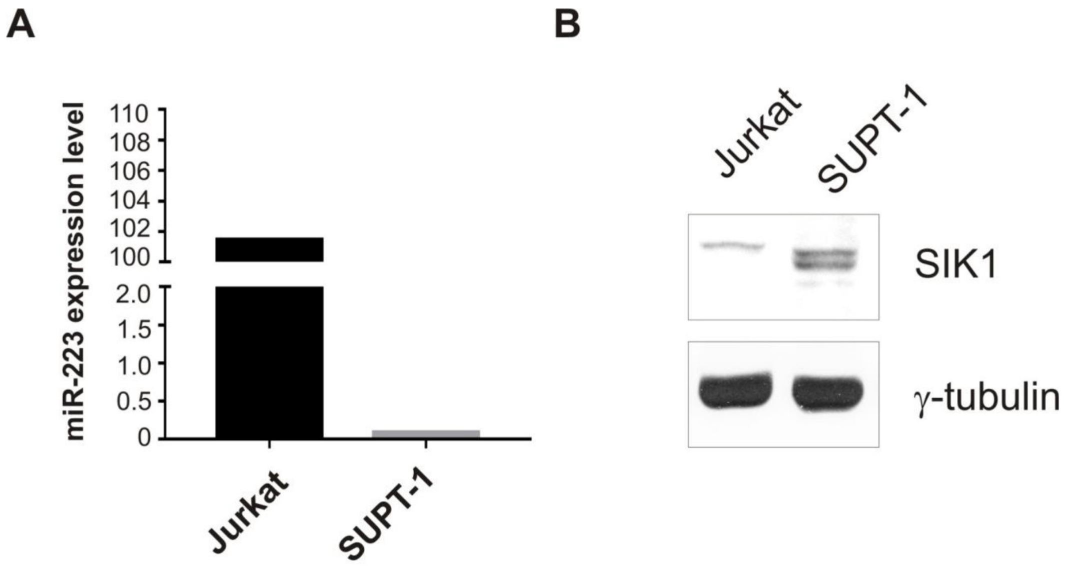

Supplementary Figure 2: Endogenous expression of miR-223 and SIK-1 in SUPT-1 and Jurkat cell lines. (A) Expression levels of miR-223 in Jurkat and SUPT-1 cell lines measured by qRT-PCR. Data have been calculated according to the comparative delta Ct method ($2^{-\Delta\Delta Ct}$), using RNU6 as endogenous control. **(B)** Western blotting analysis of SIK-1 in Jurkat and SUPT-1 cell lines. γ-tubulin was used as loading control.

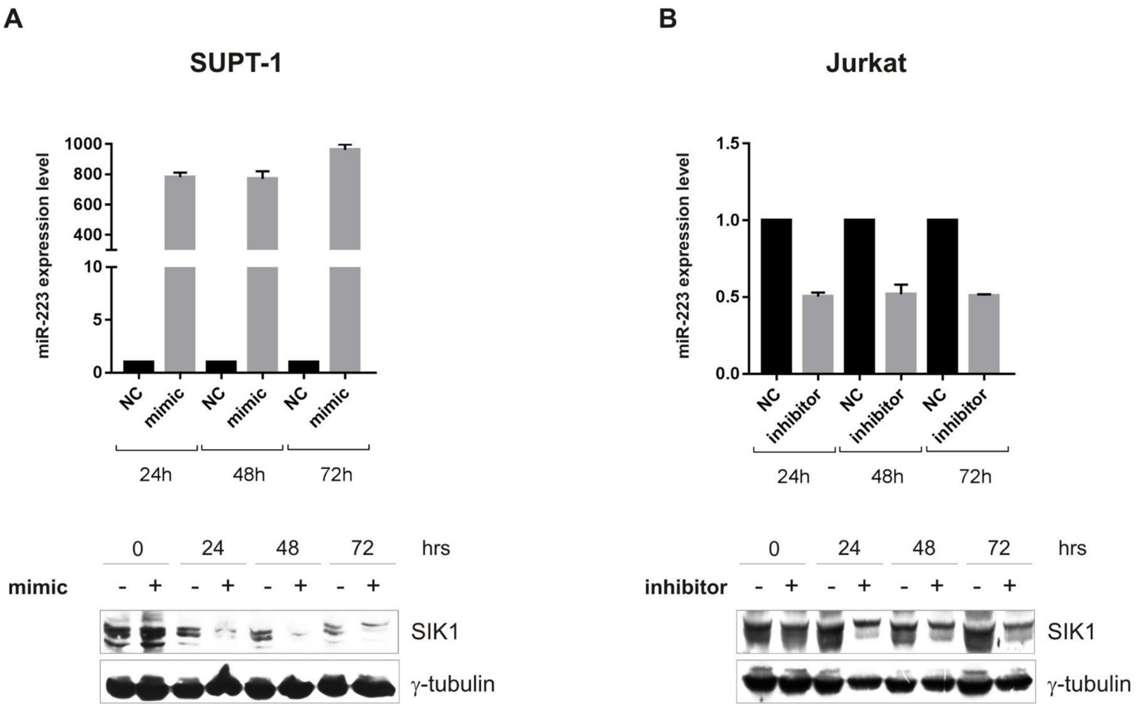

Supplementary Figure 3: Expression of miR-223 and SIK1 post-transfection of SUPT-1 and Jurkat cell lines. (A) SUPT-1 and **(B)** Jurkat were transfected with pre-miR-223 (mimic) and anti-miR-223 (inhibitor), respectively. At the top, expression levels of miR-223 in SUPT-1 and Jurkat cell lines measured by qRT-PCR at indicated post-transfection time points. Data have been calculated according to the comparative delta Ct method ($2^{-\Delta\Delta Ct}$), using RNU6 as endogenous control. Data mean ($n=3 \pm$ SEM) were compared to that of cells transfected with relative negative control (NC). Below, the Western blotting analysis of SIK-1 in SUPT-1 and Jurkat cell lines at indicated post-transfection time points; γ-tubulin was used as loading control.

```
            3' accccauaaacuguuUGACUGu 5' hsa-miR-223 (UTR1)
                              ||||||
1  GGGCAGCCCUGCAUCCUGGCACGGACACUGACUCUUACAGCAAUAACUUCAGAGGAGGUGAAGACAUCUGGCCUC 75
76 AAAGCCAAGAACUUUCUAGAAGCGAAAUAAGCAAUACGUUAGGUGUUUUGGCUUUUUAGUUUAUUUUUGUUUUAU 150
```

```
        3'accccAUAAACUGU-UUGACUgu 5' hsa-miR-223 (UTR4)
                   | |||  || ||||||
2101 UAGUUUUUUUUUUUACCAUAACUGAAUUAUUUUAUCUCUUAUGUUAACAUGAGAAAUGUAUGCCAAAUGAUUAGU 2175
2176 UGAUGUAUGUUUUUUAAUUUAAUAUUUAAAUAAAAUAUUUGGAAGGAAA 2224
```

Supplementary Figure 4: miR-223 binding sites on 3'-UTR sequence of SIK1. Two putative binding sequences, named UTR1 and UTR4, have been predicted by using on-line tool at http://www.microrna.org/. The whole UTR sequence, including portions UTR2 and UTR3 comprised between UTR1 and UTR4, has been subcloned into pmirGLO reporter plasmid and tested by luciferase assay.

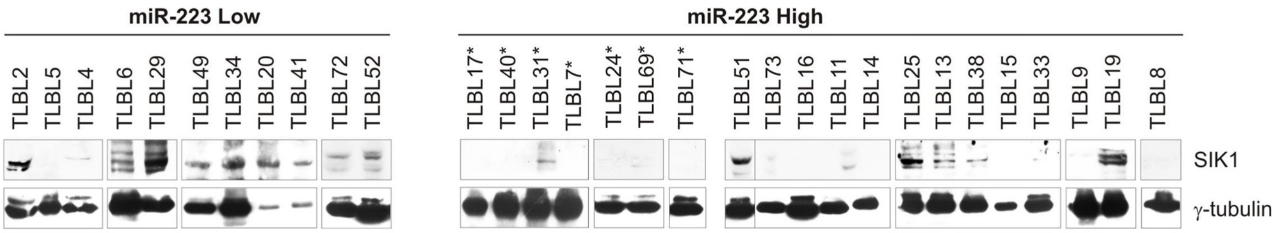

Supplementary Figure 5: SIK1 expression in T-LBL primary tumors, according to miR-223 expression levels. Vertical lines have been inserted to indicate repositioned gel lanes. The membranes were probed for γ-tubulin as loading control. Relapsed patients are indicated with *.

Supplementary Table 1: Clinical features of T-LBL clinical cohort.

See Supplementary File 1

Supplementary Table 2: List of NOTCH1 mutations identified in 35 T-LBL patients. Sequence numbering is based on GenBank accessions NM_017617.4 and NP_060087.3.

See Supplementary File 2
