# Supplementary material for: Clinical impact of miR-223 expression in pediatric T-Cell lymphoblastic lymphoma
Source: Oncotarget. 2017 Nov 11;8(64):107886–98. doi: 10.18632/oncotarget.22386 (PMC5746112; doi:10.18632/oncotarget.22386)
Supplement: Supplementary file 2 [file oncotarget-08-107886-s002.doc]

**Supplementary Table 1:** Clinical features of T-LBL clinical cohort

| **Characteristics** | **Categories** | **# Pts** | % | **# Pts** | % |
| --- | --- | --- | --- | --- | --- |
|  |  | Available tissue (n=67)* | | No available tissue (n=47)° | |
| **Age at diagnosis (years)** | < median age  > median age | 33  34 | 49  51 | 23  24 | 49  51 |
| **Gender** | Male  Female | 51  16 | 76  24 | 34  13 | 72  28 |
| **Stage** | I+II  III+IV | 7  60 | 10  90 | 7  40 | 15  85 |
| **Mediastinal involvement** | Yes  No | 52  15 | 78  22 | 31  16 | 66  34 |
| **BM involvement** | Yes  No | 10  57 | 15  85 | 9  38 | 19  81 |
| **CNS involvement** | Yes  No | 3  64 | 5  95 | 1  46 | 2  98 |

*median age: 9.5 years; ° median age: 9.3 years
