# Supplementary material for: Clinical impact of miR-223 expression in pediatric T-Cell lymphoblastic lymphoma
Source: Oncotarget. 2017 Nov 11;8(64):107886–98. doi: 10.18632/oncotarget.22386 (PMC5746112; doi:10.18632/oncotarget.22386)
Supplement: Supplementary file 3 [file oncotarget-08-107886-s003.doc]

**Supplementary Table 2: List of *NOTCH1* mutations identified in 35 T-LBL patients. Sequence numbering is based on GenBank accessions NM_017617.4 and NP_060087.3**

|  |  |  |  |  |
| --- | --- | --- | --- | --- |
|  | **HD domain** | | **PEST domain** | |
| **patient** | **DNA** | **protein** | **DNA** | **protein** |
| TLBL1 |  |  | c.7289G>C | p.G2430A |
| TLBL2 |  |  | c.7216C>T | p.Q2406^*^ |
| TLBL4 | c.4732_4734delGTG | p.V1578del |  |  |
| TLBL5 | c.4776(Ins12) | p.F1592ins4 | c.7180C>T | p.Q2394^*^ |
| TLBL6 | c.4721T>C | p.L1574P |  |  |
| TLBL9 | c.4817(Ins9) | p.F1606ins3 |  |  |
| TLBL14 | c.4721T>C | p.L1574P |  |  |
| TLBL19 | c.4793G>C | p.R1598P | c.7373C>GGA | p.L2457fs^*^19 |
| TLBL21 |  |  | c.7541-42delCT | p.S2513fs^*^3 |
| TLBL22 | c.4733T>A | p.V1578E | c.7354(insGA) | p.L2451fs^*^25 |
| TLBL24 |  |  | c.7171C>TAA | p.Q2391^*^ |
| TLBL29 | c.4847T>A | p.I1616N |  |  |
| TLBL30 |  |  | c.7397C>T | p.T2466M |
| TLBL37 | c.4816TT>GCCCTGGA | p.F1606>ALD | c.7543G>T | p.E2515^*^ |
| TLBL39 | c.4754T>C | p.L1585P |  |  |
| TLBL42 |  |  | c.7528C>T | p.L2510F |
| TLBL46 | c.4825del75 | p.R1608del25 | c.6853G>A, | p.V2285I |
| TLBL47 | c.5033T>C | p.L1678P | c.7541-42delCT | p.S2513fs^*^3 |
| TLBL48 |  |  | c.7535-36insC | p.S2513fs^*^3 |
| TLBL50 | c.4775T>C | p.F1592S |  |  |
| TLBL52 | c.4799T>C | p.L1600P | c.7606G>A | p.V2536I |
| TLBL53 | c.4757G>C | p.R1586P |  |  |
| TLBL54 | c.4775-76insAAGGAC | p.F1592>LRT |  |  |
| TLBL57 | c.5033T>C | p.L1678P |  |  |
| TLBL59 | c.5033T>C | p.L1678P |  |  |
| TLBL61 | c.4793G>C | p.R1598P |  |  |
| TLBL62 | c.4721T>C | p.L1574P |  |  |
| TLBL64 | c.4799T>C | p.L1600P |  |  |
| TLBL66 | c.4778T>C | p.L1593P | c.7606G>A | p.V2536I |
| TLBL67 | c.4793G>C | p.R1598P |  |  |
| TLBL68 | c.4721T>C | p.L1574P | c.7130-37del-ins7 | p.P2410fs^*^10 |
| TLBL69 | c.5033T>C | p.L1678P |  |  |
| TLBL70 | c.4796T>G | p.V1599G |  |  |
| TLBL72 | c.4810_4812delGTG | p.V1604del | c.7388indel | p.A2463fs^*^9 |
| TLBL74 | C.4746insGCC | p.P1582insA | c.7362ins9 | p.H2454insPPV |
